# Combination treatment of a novel CXCR3 antagonist ACT-777991 with an anti-CD3 antibody synergistically increases persistent remission in experimental models of type 1 diabetes

**DOI:** 10.1093/cei/uxad083

**Published:** 2023-07-17

**Authors:** Urs Christen, Laetitia Pouzol, Mélanie Tunis, Anna Sassi, Camilla Tondello, Monika Bayer, Edith Hintermann, Daniel S Strasser, Sabrina Schuldes, Ulrich Mentzel, Marianne M Martinic

**Affiliations:** Pharmazentrum Frankfurt, Goethe University Frankfurt, Germany; Immunology and Pharmacology Department, Idorsia Pharmaceuticals Ltd., Hegenheimermattweg 91, Allschwil, Switzerland; Immunology and Pharmacology Department, Idorsia Pharmaceuticals Ltd., Hegenheimermattweg 91, Allschwil, Switzerland; Immunology and Pharmacology Department, Idorsia Pharmaceuticals Ltd., Hegenheimermattweg 91, Allschwil, Switzerland; Pharmazentrum Frankfurt, Goethe University Frankfurt, Germany; Pharmazentrum Frankfurt, Goethe University Frankfurt, Germany; Pharmazentrum Frankfurt, Goethe University Frankfurt, Germany; Translational Biomarkers Department, Idorsia Pharmaceuticals Ltd., Hegenheimermattweg 91, Allschwil, Switzerland; Project Management Department, Idorsia Pharmaceuticals Ltd., Hegenheimermattweg 91, Allschwil, Switzerland; Pharmacology and Preclinical Development Department, Idorsia Pharmaceuticals Ltd., Hegenheimermattweg 91, Allschwil, Switzerland; Immunology and Pharmacology Department, Idorsia Pharmaceuticals Ltd., Hegenheimermattweg 91, Allschwil, Switzerland

**Keywords:** CXCR3 antagonism, ACT-777991, anti-CD3 antibody, type 1 diabetes, plasma C-peptide, combination therapy

## Abstract

Treatment of patients with recent-onset type 1 diabetes with an anti-CD3 antibody leads to the transient stabilization of C-peptide levels in responder patients. Partial efficacy may be explained by the entry of islet-reactive T-cells spared by and/or regenerated after the anti-CD3 therapy. The CXCR3/CXCL10 axis has been proposed as a key player in the infiltration of autoreactive T cells into the pancreatic islets followed by the destruction of β cells. Combining the blockade of this axis using ACT-777991, a novel small-molecule CXCR3 antagonist, with anti-CD3 treatment may prevent further infiltration and β-cell damage and thus, preserve insulin production.

The effect of anti-CD3 treatment on circulating T-cell subsets, including CXCR3 expression, in mice was evaluated by flow cytometry. Anti-CD3/ACT-777991 combination treatment was assessed in the virally induced RIP-LCMV-GP and NOD diabetes mouse models. Treatments started at disease onset. The effects on remission rate, blood glucose concentrations, insulitis, and plasma C-peptide were evaluated for the combination treatment and the respective monotherapies.

Anti-CD3 treatment induced transient lymphopenia but spared circulating CXCR3^+^ T cells. Combination therapy in both mouse models synergistically and persistently reduced blood glucose concentrations, resulting in increased disease remission rates compared to each monotherapy. At the study end, mice in disease remission demonstrated reduced insulitis and detectable plasma C-peptide levels. When treatments were initiated in non-severely hyperglycemic NOD mice at diabetes onset, the combination treatment led to persistent disease remission in all mice.

These results provide preclinical validation and rationale to investigate the combination of ACT-777991 with anti-CD3 for the treatment of patients with recent-onset diabetes.

## Teaser

Combining anti-CD3 treatment with the blockade of the CXCR3/CXCL10 axis using ACT-777991, a CXCR3 antagonist, further preserves B-cell damage and synergistically increases persistent remission in experimental models of type 1 diabetes.

## Graphical Abstract

**Figure:**
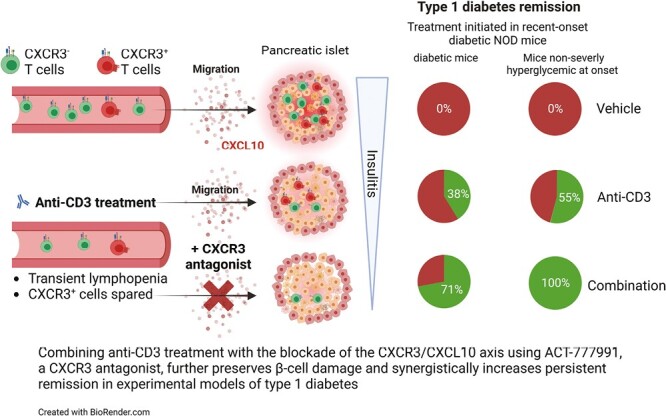

## Introduction

Type 1 diabetes is a chronic disease characterized by hyperglycemia, resulting from the progressive destruction of insulin-producing β-cells in the pancreatic islets of Langerhans. Antibodies against specific islet antigens are measurable in more than 90% of people with newly diagnosed type 1 diabetes. The high risk of progression in the presence of multiple autoantibodies has led to defining different stages of the disease. During stage 1 of type 1 diabetes, individuals present with at least two different types of diabetes-related autoantibodies. In stage 2, individuals in addition display dysglycemia but without clinical symptoms, while patients in stage 3 become clinically symptomatic [1]. Despite decades of research, no treatment exists to cure or prevent type 1 diabetes, and patients still rely on daily, lifelong insulin administration to survive. Novel therapeutic strategies aimed at blocking the autoimmune destruction of β-cells to preserve insulin production and reduce disease-related clinical complications [2]. The first FDA-approved immunotherapy targeting T-cells using an anti-CD3 monoclonal antibody (aCD3), teplizumab, was shown to delay the onset of stage 3 type 1 diabetes [3–6]. Anti-CD3 treatment also demonstrated efficacy in multiple randomized trials in patients with recent-onset type 1 diabetes, transiently preserving β cells’ insulin secretory capacity [7–9]. The mechanism underlying the efficacy of aCD3 treatment is not fully understood and was proposed to be a restoration of immune tolerance against β cells-associated antigens, allowing for an immune reset [10–14]. However, not all patients respond to the aCD3 treatment and responder patients show only a transient stabilization of C-peptide, a surrogate for β cells function [9, 15]. This transient effect suggests that the autoimmune process is insufficiently controlled by aCD3 monotherapy and that spared and/or new autoreactive T cells continue to enter pancreatic islets causing further β-cell damage. Leukocyte infiltration to pancreatic islets is mainly orchestrated by chemokines secreted by stressed β-cell, endothelial cells, and invading immune cells [16]. Evidence from both type 1 diabetes animal models and human pancreatic organ donors with recent-onset type 1 diabetes identified CXCL9 and CXCL10 as key chemokines that induce the attraction of autoreactive T cells bearing the corresponding chemokine receptor CXCR3 to pancreatic islets [16–23]. However, exclusive blockade of this chemokine axis yielded contradictory results for type 1 diabetes development and control in animal models [24–26], emphasizing the need to target several autoimmune pathways to treat this chronic disease. In line with this hypothesis, administration of an antibody neutralizing CXCL10 following aCD3 treatment resulted in reduced CD8^+^ T-cell infiltration into pancreatic islets and increased remission of diabetes compared to aCD3 monotherapy in preclinical disease models [27].

CXCR3 is increased on various T-cell subsets in patients with type 1 diabetes, including effector memory CD8^+^ T cells [28]. Given that aCD3 treatment induces transient lymphopenia affecting preferentially CD4^+^ vs. CD8^+^ T cells, both in patients with type 1 diabetes [13] and in preclinical models [27], and increases proportions of circulating antigen-experienced CD8^+^ T-cell subsets in patients with type 1 diabetes [13], we hypothesized that aCD3 treatment may spare CXCR3 autoreactive T cells. These spared T cells could then migrate into the islets along the CXCL10 concentration gradients to continue the β-cell damaging process. The objectives of the study were therefore, (i) to evaluate whether aCD3 treatment in mice replicates the CD8 T-cell subset modulation seen in humans and investigate their CXCR3 expression, and (ii) to assess whether the combination of aCD3 with the CXCR3 antagonist ACT-777991 [29] enhances type 1 diabetes control in preclinical models. The combination treatment was evaluated in the same two type 1 diabetes mouse models that had previously shown efficacy with aCD3 treatment [27], the virally induced RIP-LCMV-GP model, where transgenic mice express the glycoprotein (GP) of lymphocytic choriomeningitis virus (LCMV) under control of the rat insulin promoter (RIP) in pancreatic β-cells [30, 31], and the NOD mouse model [32].

## Methods and materials

### Mice

Female C57BL/6 mice (8–9 weeks) were purchased from Janvier Laboratories or Envigo. Generation and screening by PCR of H-2^b^ RIP-LCMV-GP transgenic mice (C57BL/6 background) were conducted as previously described [30, 33]. Female NOD mice were purchased from Charles River or Jackson Laboratories. All mice were for at least seven days before use and were group-housed under climate- and light-controlled conditions. Mice had access to food and drinking water *ad libitum*. All experimental procedures were conducted in accordance with the German animal welfare ordinance on the use of experimental animals or with the Swiss animal protection law. The animal studies were approved by the Regierungspräsidium Darmstadt, Germany or by the Basel Cantonal Veterinary Office, Switzerland.

### Test compounds

Monoclonal Armenian hamster anti-mouse CD3ε antibody (145-2C11 F(abʹ)2 fragment, pepsin digested) and the Armenian hamster F(abʹ)2 fragment isotype control were obtained from BioXCell. ACT-777991 was provided by Idorsia Pharmaceuticals Ltd and prepared as food admix in the regular food pellet 3336 (Granovit AG) at a final concentration of 0.6 mg/g food. At this selected dose, ACT-777991 was shown to inhibit the chemotaxis of CXCR3^+^ T cells to a site of inflammation *in vivo* [29]. The food pellet alone was crushed and re-pelleted for use as the vehicle treatment.

### RIP-LCMV-GP model

Male and female RIP-LCMV-GP mice (aged 6–16 weeks) were used for the experiments. To induce type 1 diabetes, mice were infected with LCMV Armstrong clone 53b on Day 0, as previously described [33]. aCD3 or isotype control was injected i.v. (3 µg/day) on Days 10, 11, and 12, as previously described [27]. ACT-777991 or vehicle (control food) treatment was initiated on Day 12 (evening) after randomization for similar blood glucose concentrations (BGCs). ACT-777991 food admix was given until Day 28 (ACT-777991_acute_) or Day 84 (ACT-777991_chronic_). Blood glucose was monitored with a dynaValeo glucometer (dynamiCARE) on Days 0 (baseline), 7, 10, 12, 14, 17, and 21 after infection, and then in weekly intervals until the end of the study (Day 84). The study design is illustrated Fig. 3A. Diabetes was defined as blood glucose concentrations (BGCs) ≥ 300 mg/dl [27]. Only mice with BGCs≥300 mg/dl and < 600 mg/dl at least once between Days 10 and 14 after LCMV infection were included in the final data evaluation. Mice with BGCs ≥ 600 mg/dl on three consecutive measurements were euthanized as per the approved animal license and the last-measured BGC was kept until the end of the study. Diabetes remission was defined as a stable reversion to BGCs < 300 mg/dl. Pancreatic tissues were collected, quick-frozen on dry ice, and cut into 7-µm sections for immunohistochemistry.

### NOD model

NOD mice were monitored weekly for BGCs, with type 1 diabetes onset defined as BGC ≥ 300 mg/dl. At disease onset, aCD3 or isotype control was injected i.v. (30 µg/day) on Days 1, 2, and 3 [27], together with ACT-777991 or vehicle treatment until the end of the study (40 weeks of age or earlier as per the approved animal license). Treatment groups were randomized for similar BGCs and age at disease onset. All NOD mice included in the efficacy experiments were used at the animal facility at the Goethe University in Frankfurt (Germany). Three cohorts of mice, obtained at different time points, were all purchased from Charles River. From all NOD mice received, approximately 60% developed type 1 diabetes within 14–30 weeks of age.

In addition, one cohort of mice purchased from the Jackson Laboratories was used at the Idorsia Pharmaceuticals Ltd animal facilities to assess the C-peptide kinetics after diabetes onset (5 out of 9 mice developed disease).

### Immunohistochemistry

Pancreatic tissues were immersed in Tissue-Tek OCT, quick-frozen on dry ice, and cut into 7-µm tissue sections. Sections were fixed in ethanol or ethanol/acetone (1:1) at –20 °C. Sections were sequentially incubated with polyclonal guinea pig anti-insulin (Agilent/Dako A0564) primary antibody and then with biotinylated goat anti-guinea pig IgG (Vector Laboratories BA-7000-1.5) secondary antibody and avidin peroxidase conjugate (Vector Laboratories). Images were acquired with an Axioscope2 microscope (Zeiss). The degree of insulitis was scored based on the criteria described in Fig. 3F.

### Flow cytometry

Female C57BL/6 mice (8–9 weeks of age) were treated with either aCD3 or isotype i.p. (3 µg/day) on Days 0, 1, and 2. Whole blood was collected at the tail vein starting 2, 24, and 48 h at 6, 9, 13, 16, 22, and 27 days after the last i.p. injection and analyzed using flow cytometry. Each mouse was bled maximally five times. At each time point, 50 µl of blood were stained with the following surface fluorochrome monoclonal anti-mouse antibodies from Biolegend: PB-CD45 (Clone 30-F11), FITC-CD8a (Clone 5H10-1), PE-Cy7-TCRβ (Clone H57-597), APC-CXCR3 (Clone CXCR3-173), APC-Cy7-CD19 (Clone 6D5), and PE anti-mouse CD4 (BD Biosciences, Clone GK1.5). To confirm the findings obtained in the kinetic experiment, a second independent experiment was performed under the same conditions except that antibody injections were done i.v. and blood was collected 24 h after the last injection. Whole blood (50 µl) was stained with the following surface fluorochrome monoclonal anti-mouse antibodies from Biolegend: BV650-CD8a (Clone 53-6.7), BV605-CD4 (Clone GK1.5), PE-Cy7-TCRβ (Clone H57-597), APC-CXCR3 (Clone CXCR3-173), AF700-CD19 (Clone 6D5), PB-CD62L (Clone MEL-14), and PerCP-Cy5.5-KLRG1 (Clone 2F1), as well as APC-Cy7-CD44 (Clone IM7) and FITC-CD69 (Clone H1.2F3) from BD Biosciences. Staining was performed on ice, in the dark, for 20 min, after preincubation with an Fc receptor blocker (CD16/CD32, BD Biosciences). After staining, red blood cells were lysed using diluted RBC lysis buffer (BioLegend). Dead cells were excluded based on positive propidium iodide staining (PI, CAS 25535-16-4, Sigma–Aldrich). Samples were acquired either on a Gallios or a CytoFLEX Flow cytometer (Beckman Coulter). Data were analyzed using Kaluza analysis software version 2.1 (Beckman Coulter). Cell subsets were quantified among singlets/viable cells. The CXCR3^+^ T cells were identified based on the fluorescence minus one control for CXCR3. The gating strategy is illustrated in Supplementary Fig. S4.

### Quantification of plasma C-peptide concentration

Whole blood from NOD mice was collected in EDTA-coated tubes (BD Microtainer), centrifuged to prepare plasma and assayed for C-peptide concentration using the mouse C-peptide ELISA kit (Crystal Chem) according to the manufacturer’s instructions.

### Statistical analysis

Statistical analysis was performed using GraphPad Prism version 9 using the tests specified in the figure legends. Statistical significance was conferred with *P* values < 0.05. Unless otherwise stated, data are expressed as means ± SEM.

## Results

### Anti-CD3 treatment induces transient lymphopenia but spares circulating central memory and effector/effector memory CD8^+^ T cells in mice

**Figure 1.**
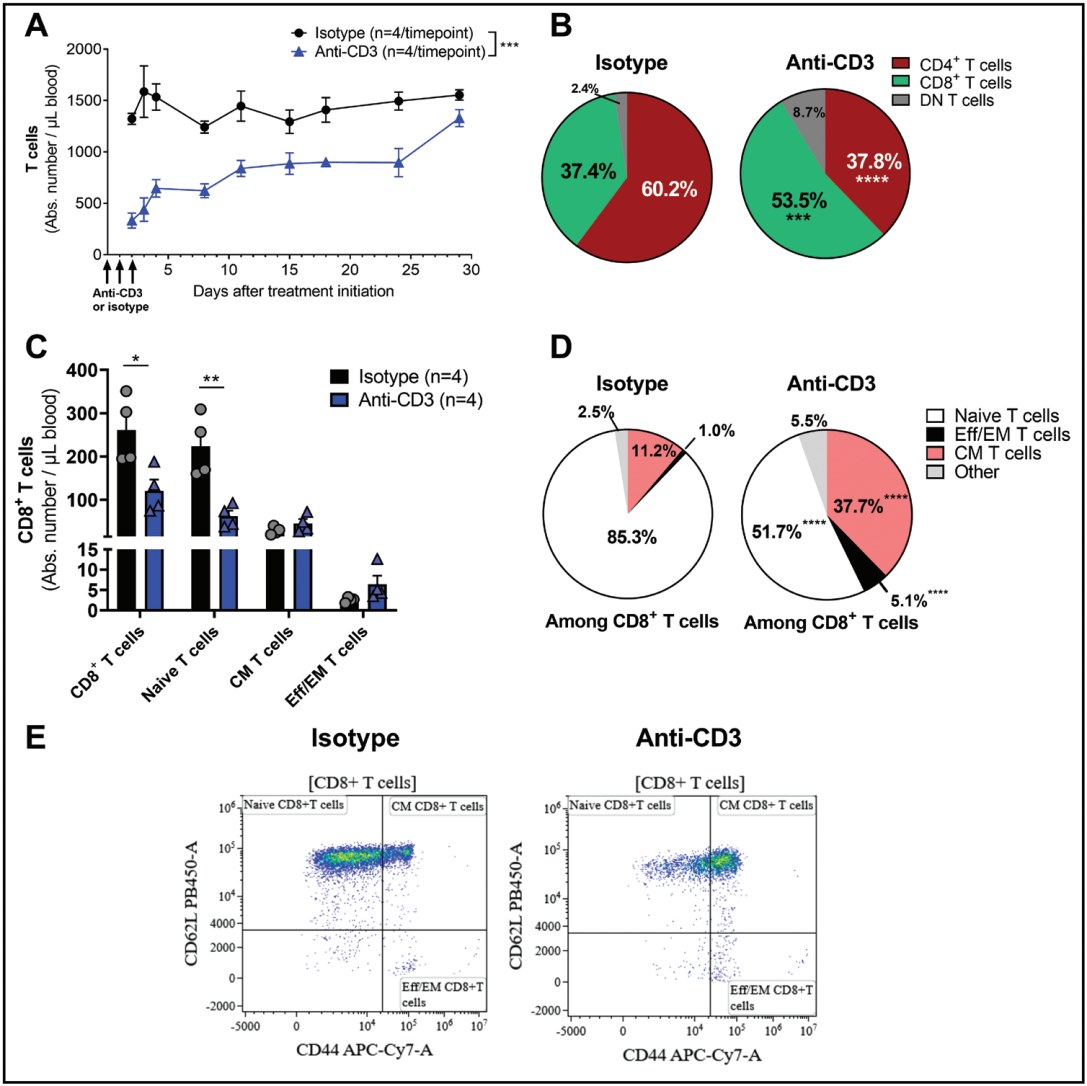
**Administration of aCD3 induces transient lymphopenia but spares circulating CD8^+^ central memory and effector/effector memory T cells, increasing their proportions in naïve mice.** C57BL/6 mice were injected on Days 0, 1, and 2 with 3 µg of anti-mouse CD3ε (triangles) or isotype (circles) antibody (*n* = 4/time point/group). Blood was collected at different time points and processed for flow cytometry analysis. Gating strategy is depicted in Supplementary Fig. S4. (**A**) Quantification of the absolute number of blood T cells. Results are expressed as mean ± SEM. (**B**) Percentage of CD4^+^, CD8^^+^^ or double-negative (DN) cells among T cells in isotype-treated mice (left diagram) versus aCD3-treated mice (right diagram), 24 h after the last injection. (**C**) Quantification of the absolute number of CD8^+^ T-cell subsets in blood, 24 h after the last injection. Results are expressed as mean ± SEM. (**D**) Percentage of naïve (CD62L^+^CD44^-^), central memory (CM, CD62L^+^CD44^+^), effector/effector memory (Eff/EM, CD62L^-^CD44^+^) or other (CD62L^-^CD44^-^) CD8^+^ T-cells among CD8^+^ T-cells in isotype-treated mice (left diagram) versus aCD3-treated mice (right diagram), 24 h after the last injection. (**E**) Representative flow cytometry plots and gating strategy for the different CD8^+^ T-cell subsets in isotype versus aCD3-treated mice. *P < 0.05, **P < 0.01, ***P < 0.001, ****P < 0.0001 versus isotype-treated mice using paired (**A**) or unpaired *t*-test (**B–D**)

In patients with type 1 diabetes, aCD3 treatment transiently reduces CD3-expressing lymphocytes and induces shifts within the CD8^+^ T-cell compartment [13]. To understand the kinetic effect of aCD3 treatment on the circulating pool of T cells in mice, three daily injections of 3 µg of aCD3 or isotype control were administered to naïve C57BL/6 mice, and T-cells subsets were quantified by flow cytometry. Treatment with aCD3 transiently reduced blood T-cell numbers over the 29-day study period, reaching a maximum reduction of 75% vs. isotype-injected mice two h after the last injection (Day 2; Fig. 1A). Treatment with aCD3 reduced more CD4^+^ than CD8^+^ T cells, reaching a maximum reduction of 85% versus 59% compared to isotype-treated mice, respectively (Day 2; Supplementary Fig. S1A–S1B). This led to an increased proportion of CD8^+^ T cells in aCD3-versus isotype-treated mice (Fig. 1B), resulting in a transient increase in the CD8:CD4 ratio (Supplementary Fig. 1C). Among CD8^+^ T cells, aCD3 treatment significantly reduced the number of naïve T cells and spared circulating central memory (CM) and effector/effector memory (Eff/EM) T cells (Fig. 1C), leading to their significantly increased proportion in blood (37.7% and 5.1%, respectively, in aCD3-treated mice versus 11.2% and 1.0% in isotype-treated mice) (Fig. 1D–E).

### Anti-CD3 treatment increases the proportion of circulating CXCR3-expressing T cells in mice

**Figure 2.**
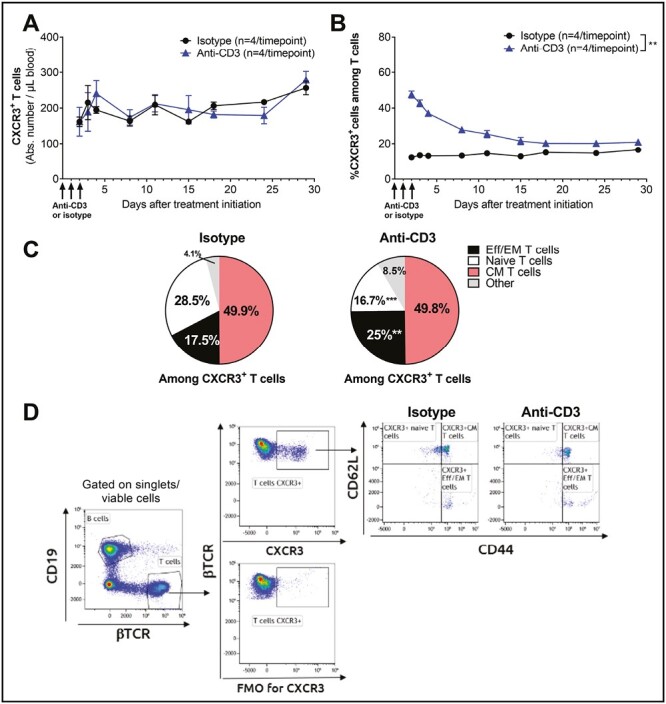
Administration of aCD3 increases the proportion of circulating CXCR3^+^ T-cells, mainly the effector/effector memory T-cell population, in naïve mice. C57BL/6 mice were injected on Days 0, 1 and 2 with 3 µg of anti-mouse CD3ε (triangles) or isotype (circles) antibody (*n* = 4/time point/group). Blood was collected at different time points and processed for flow cytometry analysis. Gating strategy is depicted in Supplementary Fig. S4. (**A**) Quantification of the absolute number of blood CXCR3^+^ T cells. Results are expressed as mean ± SEM. (**B**) Percentage of CXCR3^+^ cells among T cells in isotype and aCD3-treated mice. Results are expressed as mean ± SEM. (**C**) Percentage of naïve (CD62L^+^CD44^-^), central memory (CM, CD62L^+^CD44^+^), effector/effector memory (Eff/EM, CD62L^-^CD44^+^) or other (CD62L^-^CD44^-^) cells among CXCR3^+^ T cells in isotype-treated mice (left diagram) versus aCD3-treated mice (right diagram), 24 h after the last injection. (**D**) Representative flow cytometry plots and gating strategy for the different CXCR3^+^ T-cell subsets in isotype versus aCD3-treated mice. Gates were set based on a fluorescence minus one (FMO) control for CXCR3. **P < 0.01, ***P < 0.001 versus isotype-treated mice using paired (**A**&**B**) or unpaired *t*-test (**C**)

CXCR3 is a chemokine receptor expressed by several lymphocyte subtypes, including antigen-experienced CD8^+^ T cells. CXCR3 has been described to be responsible for T-cell migration to inflamed tissue along a CXCR3 ligand-concentration gradient [34–36]. Specifically, CXCR3 was shown to be increased on various T-cell subsets in patients with type 1 diabetes, including naïve and EM CD8^+^ T-cells and follicular T helper cells [28]. Given that aCD3 treatment increased the relative proportion of circulating CD8^+^ T cells (Fig. 1B), the effect of this treatment on CXCR3^+^ T cells was also examined. Administration of aCD3 failed to reduce absolute CXCR3^+^ T-cell numbers (Fig. 2A), leading to a significantly increased proportion of these cells in the blood (Fig. 2B). This increased proportion of CXCR3^+^ cells within the CD4^+^ T-cell population returned to control levels by Day 8 after the first aCD3 injection, while it lasted at least 29 days among CD8^+^ T cells (Supplementary Fig. 1D–1E), suggesting that aCD3 treatment preferentially spares CD8^+^ CXCR3^+^ T cells. Within the pool of circulating CXCR3^+^ T cells, aCD3 treatment did not affect the proportion of CM T-cells but significantly reduced the proportion of naïve T-cells and increased the frequency of Eff/EM T cells (Fig. 2C–2D), suggesting that these antigen-experienced T cells could still potentially be attracted to an inflamed organ such as the pancreas.

### Combination therapy of the CXCR3 antagonist ACT-777991 and aCD3 improves type 1 diabetes remission over aCD3 monotherapy in a virus-induced diabetes model

**Figure 3.**
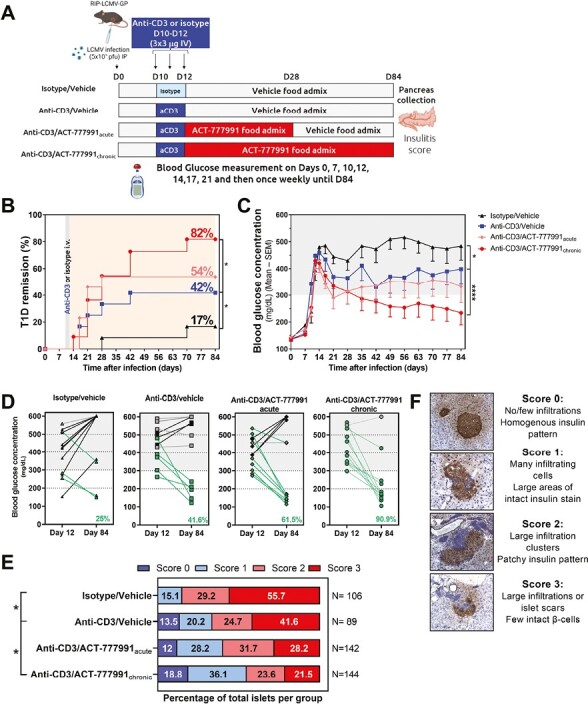
Combination of aCD3 and chronic ACT-777991 treatment increases type 1 diabetes remission compared to aCD3 monotherapy in RIP-LCMV-GP mice. (**A**) RIP-LCMV-GP mice were infected with 5 × 10^3^ plaque-forming units (pfu) LCMV intraperitoneally (IP) on Day 0. On Day 10 after infection, mice were injected daily with isotype or anti-mouse CD3ε (aCD3) (3 µg/day) intravenously (IV) for 3 days. After the last injection (Day 12), mice were treated either with vehicle food admix (Isotype/Vehicle; black triangle; *n* = 12 or aCD3/Vehicle, blue square, *n* = 12) or with ACT-777991 food admix at 0.6 mg/g of food until Day 28 (aCD3/ACT777991_acute,_ pink diamond, *n* = 13) or until the end of the study on Day 84 (aCD3/ACT777991_chronic_, red circle, *n* = 11). Only diabetic mice (BGC ≥ 300 mg/dl at least once between Days 10 and 14 after LCMV infection) were included in the analysis. Mice with BGC ≥ 600 mg/dl (fulminant diabetic mice) between Days 10 and 14 were excluded. Diabetic values were defined as BGCs ≥ 300 mg/dl (gray shading in graphs). The study design was created with BioRender.com. (**B**) Percentage of remission from type 1 diabetes (T1D), defined as % of mice reaching stable reversion of BGCs < 300 mg/dl until the end of the study (Day 84). *P < 0.05 versus aCD3/vehicle group using Cox proportional hazards regression test. (**C**) BGCs were measured at regular intervals throughout the study. Data are shown as mean BGC—SEM. *P < 0.05, ****P < 0.0001 versus aCD3/vehicle group using two-way ANOVA followed by Tukey’s multiple comparison test. (**D**) Comparisons of BGCs at Day 12 versus Day 84 after infection. Mice that demonstrated an increase or decrease ≥50 mg/dl at Day 84 are displayed in black or green, respectively. Mice that presented an unchanged BGC concentration (< 50 mg/dl) are shown in gray. The percentage of mice that improved from Day 12 to Day 84 is indicated in green in the bottom right corner. (**E**) Mice were sacrificed upon reaching euthanasia criteria based on their BGCs or at the end of the study (Day 84). The degree of insulitis was scored as indicated for ≥89 islets for each treatment group and data are shown as percentage of total islets evaluated. *P < 0.05 versus aCD3/Vehicle group using Kruskal–Wallis test followed by Mann Whitney *U* Test (with continuity correction). (**F**) Representative images of individual islets of Langerhans stained for insulin (brown) and counterstained with hematoxylin (blue), as well as criteria used to define the insulitis score

Blocking CXCR3 with ACT-777991 has been shown to inhibit both the migration of *ex vivo*-activated human and mouse T cells toward a CXCR3 ligand and the chemotaxis of CXCR3^+^ T cells to a site of inflammation *in vivo* [29]. To evaluate the effects of combining ACT-777991 with aCD3, diabetic RIP-LCMV-GP mice were injected with three daily doses of 3 µg aCD3 or isotype control on Days 10–12 after LCMV infection, followed by acute (Days 13–28) or chronic ACT-777991 treatment (Days 13–84) (Fig. 3A). In line with published data [27], aCD3 treatment resulted in remission of disease (stable reversion to BGCs < 300 mg/dl) in 42% (5 of 12) diabetic mice versus 17% (2 of 12) of isotype-treated mice (Fig. 3B). In the chronic setting, aCD3/ACT-777991 further increased the therapeutic benefit over aCD3 monotherapy, resulting in a disease remission rate of 82% (9 of 11; Fig. 3B). The therapeutic benefit conferred by the combination therapy was also reflected in a significant decrease of mean BGCs in aCD3/ACT-777991_chronic_ versus aCD3 monotherapy groups (234 mg/dl versus 398 mg/dl on Day 84, *P* < 0.0001, respectively; Fig. 3C). When comparing individual BGCs from Day 12 (last aCD3 injection) versus the end of the study (Day 84), 90.9 % of mice in the aCD3/ACT-777991_chronic_ group showed a reduced BGC and 64% of diabetic mice in this group reverted to BGC < 200 mg/dl (Fig. 3D). Mice in the aCD3 monotherapy group displayed a mixed outcome, with 41.6% of mice presenting a BGC decrease from Day 12 versus Day 84, while the remaining mice presented with a BGC increase or stabilization (Fig. 3D). In the acute setting, aCD3/ACT-777991 tended to increase diabetes remission rate versus aCD3 monotherapy as also shown by a higher frequency of animals showing improved BGC at the end of the study (8 of 13 [61.5%] vs. 5 of 12 [41.6%]) (Fig. 3B–D). Nevertheless, the additional improvement in remission rate seen with the chronic treatment, emphasizes the need for sustained CXCR3 axis inhibition to maximize the therapeutic benefit over aCD3 monotherapy. Next, the pancreas was assessed for the degree of insulitis at end-of-study. Histopathological evaluation demonstrated that aCD3 monotherapy had a significant effect on insulitis compared to isotype-treated animals where most islets were massively infiltrated with immune cells, leaving behind islets scars (Fig. 3E–F). Importantly, aCD3/ACT-777991_chronic_ treatment was superior to aCD3 monotherapy, as shown by a significant reduction in insulitis compared to the aCD3 monotherapy group (Fig. 3E–F), supporting the enhanced disease remission in this treatment group. Of note, the superior efficacy observed in the aCD3/ACT-777991_chronic_ group was not due to a potential effect of ACT-777991 on LCMV clearance as the viral load, both in the spleen and liver, was not different between infected mice given control food and mice treated with ACT-777991 (Supplementary Fig. S2).

### Combination therapy of the CXCR3 antagonist ACT-777991 with aCD3 synergistically reverts type 1 diabetes in NOD mice

**Figure 4.**
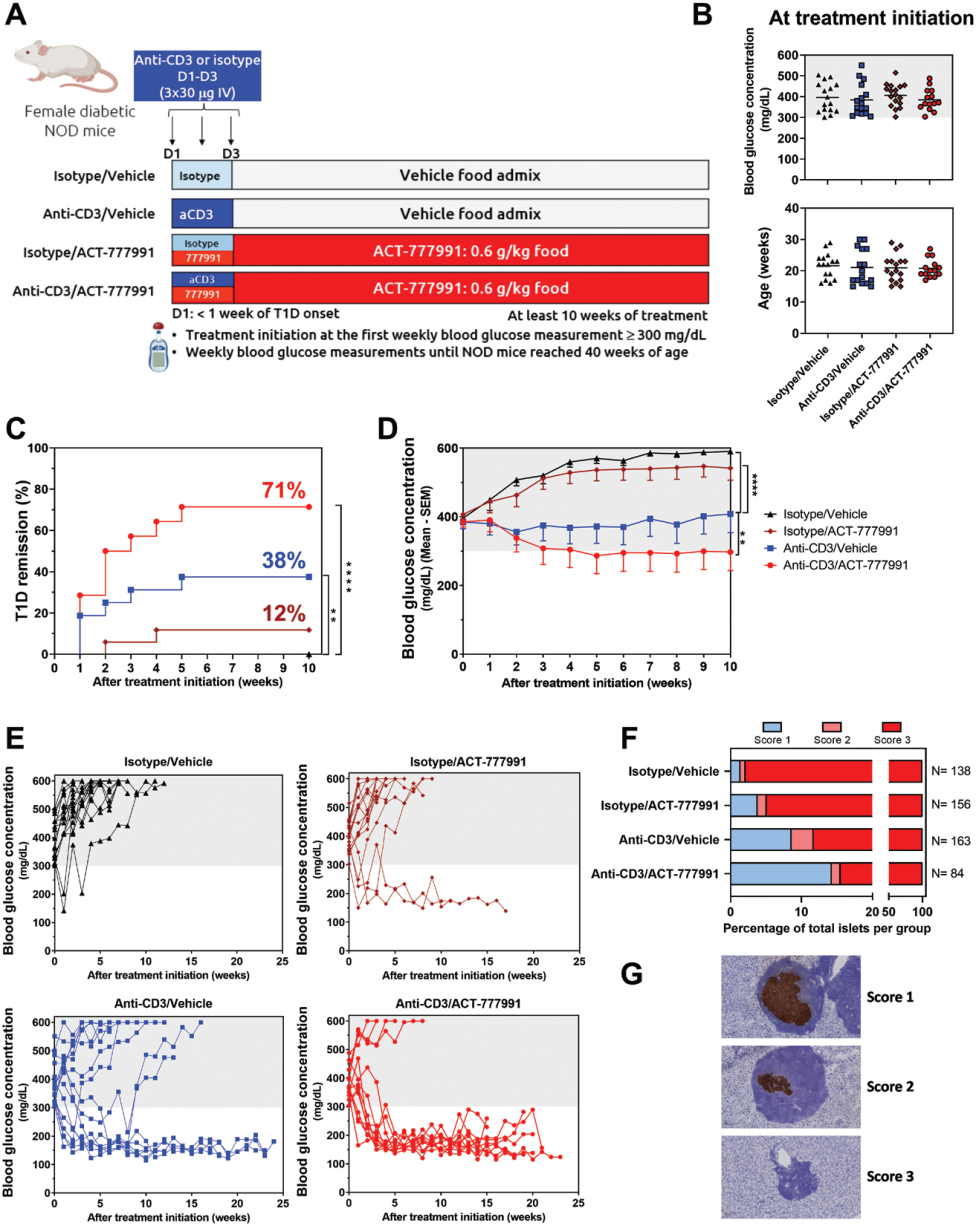
Combination of aCD3 and chronic ACT-777991 treatment increases type 1 diabetes remission compared to aCD3 monotherapy in NOD mice. Female NOD mice were monitored weekly for blood glucose levels. At the first blood glucose measurement fulfilling the diabetic criterion of BGC ≥ 300 mg/dL, diabetic NOD mice initiated treatment. (**A**) Mice were injected daily with isotype or anti-mouse CD3ε (aCD3) (30 µg/day) intravenously (IV) for three days (D1–D3). In parallel, mice received vehicle or ACT-777991 food admix (0.6 mg/g food) until the end of the study. Mice were randomized in four treatment groups based on their BGC and age at disease onset: (1) Isotype antibody together with vehicle food admix (Isotype/Vehicle; triangle, *n* = 16); (2) aCD3 together with vehicle food admix (Anti-CD3/Vehicle, square, *n* = 16); (3) Isotype antibody together with ACT-777991 (Isotype/ACT-777991, diamond, *n* = 17); (4) aCD3 together with ACT-777991 (Anti-CD3/ACT-777991, circle, *n* = 14). All mice were treated for at least 10 weeks; the end of the study was reached when mice were 40 weeks of age. Diabetic values were defined as BGCs ≥ 300 mg/dL (gray shading in graphs). The study design was created with BioRender.com. (**B**) BGCs and age at treatment initiation. (**C**) Percentage of remission from type 1 diabetes (T1D), defined as % of mice reaching stable reversion of BGCs < 300 mg/dL until the end of the 10-week treatment period. **P < 0.01, ****P < 0.0001 versus Isotype/Vehicle group using Cox proportional hazards regression test. (**D**) Mean BGCs of NOD mice after treatment initiation. Data are shown as mean BGC—SEM. **P < 0.01 ****P < 0.0001 versus Anti-CD3/Vehicle group using one-way ANOVA followed by Dunnett’s multiple comparison test. (**E**) Individual BGCs over the whole study after treatment initiation. (**F**) Mice were sacrificed upon reaching euthanasia criteria based on their BGCs or at the end of the study. The degree of insulitis was scored as indicated for ≥ 84 islets for each treatment group and data are shown as percentage of total islets evaluated. (**G**) Representative images of individual islets of Langerhans stained for insulin (brown) and counterstained with hematoxylin (blue), as well as criteria used to define the insulitis score

The therapeutic benefit of aCD3/ACT-777991_chronic_ treatment observed in RIP-LCMV-GP mice was also investigated in the NOD mouse model. Recent-onset NOD mice received the combination treatment, the respective monotherapies, or isotype control (Fig. 4A). Treatment was initiated for each mouse at diabetes onset (within 1 week of measuring a BGC ≥ 300 mg/dl), between 15 and 30 weeks of age. All mice were treated for at least 10 weeks. Treatment groups were randomized for similar BGCs and age at disease onset (Fig. 4B).

In line with published studies [27], aCD3 monotherapy significantly reversed diabetes compared to isotype-treated mice (6 of 16 [38%] vs. 0 of 16 [0%]). Treatment with aCD3/ACT-777991 combination further improved the outcome resulting in a diabetes remission rate of 71% (10 of 14) (Fig. 4C). In contrast, ACT-777991 monotherapy did not result in a significant increase in diabetes remission rate compared to isotype-treated mice (Fig. 4C). The therapeutic benefit of aCD3/ACT-777991 combination treatment was further reflected in significantly reduced mean BGCs compared to aCD3 monotherapy (Fig. 4D). In addition, in all groups, mice in remission after 10 weeks of treatment persistently stayed below the diabetic threshold (<300 mg/dl) until 40 weeks of age (Fig. 4E). Furthermore, plasma C-peptide was detectable only in mice with BGCs < 300 mg/dl at end-of-study, confirming the preservation of functional β-cells in those mice (Supplementary Fig. S3). Histopathological evaluation of the pancreas further supported the superior efficacy of the combination treatment, as shown by reduction in insulitis compared to the aCD3 monotherapy group (Fig. 4F–G).

### Initiation of aCD3/ACT-777991 combination treatment prior to the development of severe hyperglycemia leads to the cure of diabetes in NOD mice

**Figure 5.**
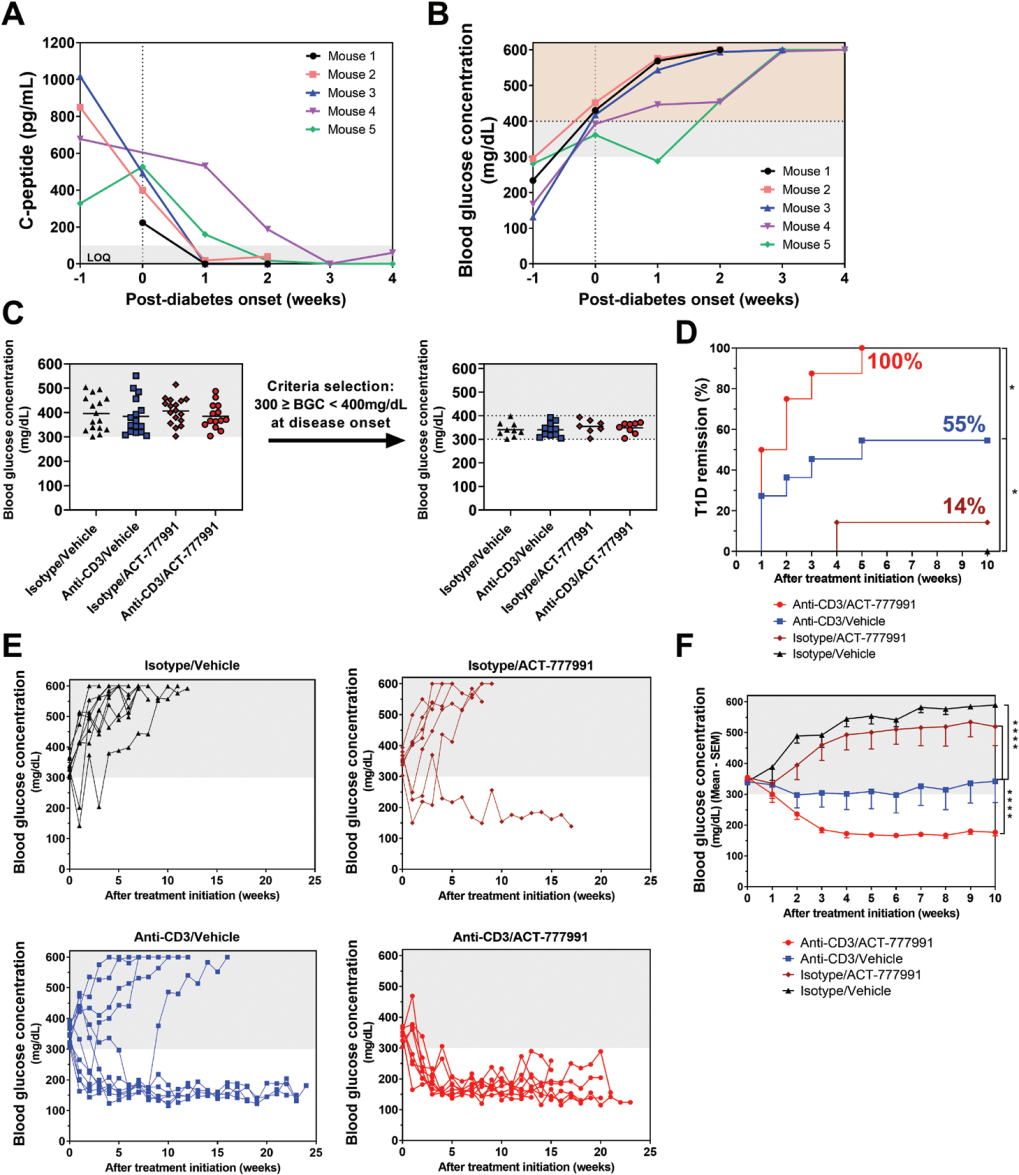
Initiation of aCD3/ACT-777991 combination therapy in non-severely hyperglycemic NOD mice at disease onset leads to the cure of diabetes. (**A and B**) Five female NOD mice were monitored weekly for blood glucose levels. At the first blood glucose measurement fulfilling the diabetic criterion of BGC ≥ 300 mg/dl, diabetic NOD mice were bled weekly for plasma C-peptide concentration measurements. Diabetic values were defined as BGCs ≥ 300 mg/dl (gray shading). (A) C-peptide plasma concentration kinetics in NOD mice after diabetes onset. Values in the gray shading are below the limit of quantification (LOQ) (≤ 100 pg/ml). (B) BGC kinetics in NOD mice after diabetes onset. The gray shading corresponds to non-severely hyperglycemic values (300 ≤ BGC < 400mg/dl) and the orange shading to severe hyperglycemic values (> 400 mg/dl). (**C**) Only diabetic NOD mice with a BGC < 400 mg/dl at disease onset/treatment initiation were included in this sub-analysis of the data from the NOD study described in Fig. 4: (i) Isotype antibody together with vehicle food admix (Isotype/Vehicle; black triangle, *n* = 9); (ii) aCD3 together with vehicle food admix (Anti-CD3/Vehicle, blue square, *n* = 11); (iii) Isotype antibody together with ACT-777991 food admix (Isotype/ACT-777991, brown diamond, *n* = 7); (iv) aCD3 together with ACT-777991 food admix (anti-CD3/ACT-777991, red circle, *n* = 8). All mice were treated for at least 10 weeks and the end of the study was reached when mice were 40 weeks of age or met the predefined euthanasia criteria. (**D**) Percentage of remission from type 1 diabetes, defined as % of mice reaching stable reversion of BGCs < 300 mg/dl until the end of the 10 weeks treatment period. *P < 0.05 versus anti-CD3/Vehicle group using Cox proportional hazards regression test. (**E**) Individual BGCs over the whole study period after treatment initiation in each treatment group. (**F**) Mean BGCs after treatment initiation. Diabetic values were defined as BGCs ≥ 300 mg/dl (gray shading). Data are shown as mean BGC—SEM; ****P < 0.0001 versus aCD3/vehicle group using one-way ANOVA followed by Dunnett’s multiple comparison test

To understand the kinetics of plasma C-peptide decline, untreated NOD mice were bled weekly from disease onset until reaching the predefined euthanasia criteria based on their BGCs. Plasma C-peptide levels declined rapidly and reached undetectable concentrations within 1–3 weeks following diabetes onset (Fig. 5A), which coincided with the measurement of BGCs > 500 mg/dl (Fig. 5B). Interestingly, mice with a BGC > 400 mg/dl at disease onset were the ones showing the fastest decline in plasma C-peptide levels (less than a week), suggesting a dramatic loss of β cells shortly after diabetes development (Fig. 5A–B).

Based on this observation, the efficacy of the combination treatment observed in the NOD study (Fig. 4) was reassessed in a post hoc analysis, this time including only non-severely hyperglycemic NOD mice at diabetes onset (BGCs between 300 and 400 mg/dl; Fig. 5C). This excluded mice which, at treatment initiation, might not have had enough functional β cells left to enable diabetes remission. With this more stringent inclusion criterion, diabetes remission increased to 55% with aCD3 monotherapy (6 of 11 mice). In contrast, the combination therapy led to full disease remission in all mice (8 of 8 [100%]) (Fig. 5D) and the BGCs remained below the diabetic threshold until end-of-study (Fig. 5E). The synergistic efficacy conferred by the combination therapy was also reflected by a significant reduction of mean BGCs reaching a normoglycemic value (173.5 mg/dl) compared to hyperglycemic mean BGCs in the aCD3 monotherapy arm (342.2 mg/dl; Fig. 5F).

## Discussion

Managing type 1 diabetes relies on daily, lifelong insulin administration and careful monitoring of dietary regimens and BGCs. Many patients eventually suffer from cardiovascular complications, impacting their life expectancy [37]. The recent approval of teplizumab, a human aCD3 monoclonal antibody, to delay the onset of stage 3 type 1 diabetes in patients who currently have stage 2 type 1 diabetes, has been a turning point in the treatment and management of this incurable disease. While teplizumab delays the progression to stage 3 diabetes, it does not prevent nor cure the disease, suggesting that the autoimmune process is not fully controlled by aCD3 monotherapy. In this context, combining aCD3 with a drug having a different mechanism of action may lead to improved disease control over each monotherapy. In the present study, the combination of ACT-777991, a new, potent, insurmountable, and selective small molecule CXCR3 antagonist [29], with aCD3 demonstrated superior efficacy over each monotherapy in persistently reverting diabetes in two different type 1 diabetes mouse models.

Teplizumab induces transient lymphopenia and remodeling in T-cell subtypes, including an increased frequency of CD8^+^ CM T cells in patients with new-onset type 1 diabetes [13]. Similar to observations made in patients, aCD3 treatment led to transient lymphopenia with a relative increase in CD8^+^ CM and Eff/EM T cells in mice, suggesting good translation of the effects to humans. The transient nature of the aCD3 treatment effect observed in responder patients and the lack of efficacy in non-responder patients might be explained by the escape and/or new generation of autoreactive T cells. CXCL10 and its cognate receptor CXCR3 have been previously identified as potential therapeutic targets in type 1 diabetes, orchestrating the migration of islet-specific T cells into the pancreas [17, 38]. For example, administration of a CXCL10-neutralizing antibody following aCD3 treatment resulted in reduced CD8^+^ T cell infiltration into pancreatic islets and increased diabetes remission compared to aCD3 monotherapy in preclinical type 1 diabetes models [27]. Our murine studies demonstrated that CXCR3-expressing T cells are spared by aCD3 treatment, increasing their proportion in blood. Among these CXCR3^+^ T-cells, aCD3 treatment reduced and increased the proportions of naïve and Eff/EM T cells, respectively, suggesting that these cells can still be attracted toward CXCR3 ligands, including CXCL10, to the inflamed pancreas. This effect could explain the transient effect of aCD3 monotherapy and, depending on the degree of escape, also the lack of response in a subgroup of patients. Even if CXCR3 was shown to be increased on various T-cell subsets in patients with type 1 diabetes, thus potentially having a detrimental effect on disease progression [28], some studies have reported reduced CXCR3 expression on blood T cells in individuals with long-lasting type 1 diabetes [39]. The conflicting results may rely on various factors such as differences in disease stages and methodologies used for CXCR3 expression assessment. Further evaluation of the function of these CXCR3-expressing T cells after aCD3 treatment would be needed to definitively establish their role in diabetes pathogenesis.

aCD3 was used at doses leading to partial efficacy, translating to remission rates of 42% and 38% in the RIP-LCMV-GP and NOD mice with recent-onset type 1 diabetes, respectively. This partial efficacy is comparable to the reported results from the AbATE teplizumab trial in patients with new-onset type 1 diabetes, where approximately 45% of the drug-treated subjects were classified as responders, defined as patients who lost < 40% of baseline C-peptide two years after the first teplizumab cycle [9]. In the RIP-LCMV-GP and NOD mouse models, the combination therapy of aCD3 with chronic ACT-777991 treatment-induced disease remission in 82% and 71% of diabetic mice, respectively. The remission rate observed with the combination therapy was superior to aCD3 or ACT-777991 monotherapies and was further supported by reduced insulitis and an increased proportion of mice with endogenous insulin production, as shown by detectable plasma C-peptide concentrations. In the virus-inducible diabetes model, early discontinuation of ACT-777991 on Day 28 after infection showed a trend in improving the remission rate compared to aCD3 monotherapy, but to a lesser extent as compared to the chronic setting, suggesting the benefit of continuous blockade of the CXCR3 axis to revert diabetes. In line with these results, the CXCR3 ligand CXCL10 has been described to be induced very early after LCMV infection and nearly all LCMV-specific CD8^+^ T cells express CXCR3 in this model [40], suggesting that chronic ACT-777991 treatment is beneficial to continuously prevent the (re)entry of autoreactive T cells into pancreatic islets. The nature of these islet-autoreactive T cells can be manifold, for example, they may have escaped aCD3 treatment and/or may include autoreactive T cells generated after the antibody was cleared. Recent evidence suggests that β-cell-specific T cells enter the pancreas from a specific stem-like autoimmune progenitor population, resident in the pancreatic lymph nodes [41]. Further evaluation of this specific population, especially after aCD3 treatment, would be of interest to determine whether this population is spared by the treatment, expresses CXCR3, and whether this progenitor pool could be targeted by the combination of aCD3 and CXCR3 antagonist.

Blockade of CXCR3 with ACT-777991 monotherapy was not efficacious in inducing diabetes remission in both mouse models versus isotype-treated animals (data not shown for the RIP-LCMV-GP model). These results are consistent with published data evaluating the impact of another small-molecule CXCR3 antagonist or CXCR3-deficient mice in the virus-induced diabetes model [25, 26]. A potential explanation could be that in the absence of an immune reset, as induced by aCD3, exclusive blockade of the CXCR3 axis, at least in mice, does not sufficiently interfere with the immune cell infiltration process into pancreatic islets and, hence, cannot inhibit the rapid progression of β-cell damage following diabetes onset. In line with these results, treatment with ACT-777991 did not impact LCMV clearance, a process involving virus-specific CXCR3^+^ CD8^+^ T cells [35, 42]. These results suggest that while the combination of ACT-777991 with aCD3 is optimal to provide superior control of the autoimmune process, ACT-777991 is not expected to detrimentally affect the immune response to a viral threat.

The therapeutic window relative to the initiation of immunomodulatory compounds is critical for preserving β-cell function and, thus, impacting diabetes progression. At diagnosis, most individuals with type 1 diabetes are believed to retain some level of functional β cells, as indicated by the presence of circulating C-peptide and supported by histological analysis of new-onset patients. In the present study, a fast decline of C-peptide, within less than a week, was observed in NOD mice with a severe hyperglycemic value (BGC ≥ 400 mg/dl) at disease onset. In contrast, diabetic mice with a lower BGC at onset had a slower plasma C-peptide decline, suggesting that those mice still retained some level of functional β cells providing an intervention window for immunomodulatory compounds to prevent further damage. In line with this hypothesis, the combination treatment was even more efficacious when initiated in non-severely hyperglycemic NOD mice at diabetes onset. While only 55% of the aCD3 monotherapy-treated diabetic mice were in remission at end-of-study, all mice treated with the aCD3/ACT-777991 combination were cured 5 weeks after treatment initiation. This complete resolution of disease was also reflected in the maintenance of normoglycemic mean BGCs across the study reaching a mean BGC of 172 mg/dl at end-of-study compared to a mean hyperglycemic BGC of 354 mg/dl in the aCD3 monotherapy group.

In conclusion, our study demonstrated that combination of the CXCR3 antagonist ACT-777991 with aCD3 treatment resulted in synergistic diabetes remission in two preclinical models of type 1 diabetes. Both drugs have distinct immunomodulatory properties that appear to act in a complementary manner, resulting in increased efficacy. Specifically, the combination treatment is expected to (i) physically and functionally deplete autoreactive T cells and reduce the islet pro-inflammatory environment [9, 11, 12] and (ii) block CXCR3 ligands-dependent migration of autoreactive CXCR3^+^ T cells to the pancreatic islets. Of note, treatment with the CXCR3 antagonist is expected to also target CXCR3^+^ immune cells other than T cells—such as NK cells, B cells, and antigen-presenting cells [36], which can all contribute to the autoimmune process—thus further reducing the autoimmune insult. Consequently, β-cell function is preserved, maximizing and extending the therapeutic benefit of aCD3 monotherapy. These encouraging preclinical data suggest that the clinical benefit observed with teplizumab in patients with recent-onset stages 3 and 2 type 1 diabetes [4, 9] might be enhanced when combined with ACT-777991.

## Supplementary Material

uxad083_suppl_Supplementary_Materials

## Data Availability

Original data are available upon request to the corresponding authors.

## Abbreviations

aCD3: anti-CD3 monoclonal antibody
BGCs: blood glucose concentrations
GP: glycoprotein
LCMV: lymphocytic choriomeningitis virus
RIP: rat insulin promoter
