# Supplementary material for: Combination treatment of a novel CXCR3 antagonist ACT-777991 with an anti-CD3 antibody synergistically increases persistent remission in experimental models of type 1 diabetes
Source: Clin Exp Immunol. 2023 Jul 17;214(2):131–43. doi: 10.1093/cei/uxad083 (PMC10714188; doi:10.1093/cei/uxad083)
Supplement: uxad083_suppl_Supplementary_Materials [file uxad083_suppl_supplementary_materials.docx]

# Supplementary Methods

***LCMV-plaque assay***

Female C57BL/6 mice (8 weeks of age) were treated with control food or ACT-777991 food admix (n=5 per group). Treatment continued until end-of-study. Five days after initiation of treatment, mice were infected with 5 × 10^3^ pfu lymphocytic choriomeningitis virus (LCMV) in 100 μl PBS intraperitoneally. After 3, 7, or 14 days, spleens and livers were removed, snap frozen in liquid nitrogen, and stored at –80 °C until further use. Frozen organs were homogenized and mixed in a dilution series with MC57 cells (C57BL/6 fibroblast cell line) and plated in 24-well plates. After a 4-h incubation period to let the cells form a monolayer, 400 μL of a 1:1 mixture of 2 × DMEM and 2% methylcellulose (MC) was added to each well. After 2 days at 37 °C, the MC overlay was removed and the cells were fixed with 4% formalin-PBS (30 min, room temperature). Formalin was removed and cells were incubated with 1% Triton X (20 min, room temperature), followed by a 60 min blocking step with 10% FCS. LCMV plaques were visualized with a rat anti-LCMV antibody (VL-4) in 1% FCS in PBS (60 min, room temperature), followed by incubation with an HRP-conjugated anti-rat IgG secondary antibody (60 min, room temperature) and with DAB as substrate (5–10 min, room temperature until color developed). Plates were dried, plaques counted, and the titers calculated according to the following formula:

$$Titer= \frac{\left( h \times10 \right)+l}{2}\times\left( dilution factor of lower dilution \right)\times5 [\frac{pfu}{mL}]$$

h = number of foci in the well with the higher dilution (fewer foci)

l = number of foci in the well with the lower dilution (more foci)

× 5 = 200 µl were added; therefore /200 × 1000

# Supplementary Figures and Figure Legends

**Supplementary Figure 1**: **Administration of aCD3 reduces blood CD4^+^ and CD8^+^ T-cells but spares circulating CXCR3^+^ T-cells, increasing their proportions among both CD4^+^ and CD8^+^ T-cells in naïve mice.** C57BL/6 mice were injected intraperitoneally on Days 0, 1, and 2 with 3 µg of anti-mouse CD3ε (blue triangles) or with isotype antibody (dark circles) (n=4/time point/group). Blood was collected at different time points and processed for flow cytometry analysis. Quantification of the absolute number of blood CD4^+^ T-cells (A) and blood CD8^+^ T-cells (B). Treatment with aCD3 increases CD8:CD4 ratio (C) and percentage of CXCR3^+^ cells among blood CD4^+^ T-cells (D) and blood CD8^+^ T-cells (E) versus isotype-treated mice. Results are expressed as mean ± SEM. *p<0.05, ***p<0.001, ****p<0.0001 using paired t test.

**Supplementary Figure 2: Treatment with ACT-777991 does not impact LCMV clearance in C57BL/6 mice.**

Lymphocytic choriomeningitis virus (LCMV) titer kinetics in the spleen and liver of C57BL/6 mice are shown as individual data (black circles [Day 3] or squares [Day 7]) and group means (vertical bars) ± standard error of the mean (SEM) (error bars). Treatment with ACT-777991 food admix (0.6 mg/g food) (991; red bars) or vehicle food (Veh; grey bars) was initiated on Day –5 and continued until the end of the study (Day 14). On Day 0, mice were infected intraperitoneally with 5 × 10^3^ pfu LCMV-Armstrong. On Days 3, 7, and 14 post-LCMV infection, mice were sacrificed (n=5 per group and time point), and spleens and livers were harvested for the evaluation of LCMV viral titers. The dotted line represents the assay’s reliability threshold.

**Supplementary Figure 3**: **Only mice in type 1 diabetes remission have detectable plasma C-peptide levels.** Female non-obese diabetic (NOD) mice were monitored weekly for blood glucose levels. At the first blood glucose measurement fulfilling the diabetic criterion of blood glucose concentration (BGC) ≥ 300 mg/dL, diabetic NOD mice received 1 of 4 treatments: (1) Isotype antibody together with vehicle food admix (Isotype/Vehicle; black triangle, n=9); (2) aCD3 together with vehicle food admix (Anti-CD3/Vehicle, blue square, n=11); (3) Isotype antibody together with ACT-777991 (Isotype/ACT-777991, brown diamond, n=9); (4) aCD3 together with ACT-777991 (Anti-CD3/ACT-777991, red circle, n=9). All mice were treated for at least 10 weeks; end-of-study was reached when mice either fulfilled the criteria for euthanasia or turned 40 weeks of age. Blood was collected to measure BGCs (A) and plasma C-peptide concentrations (B). Data are shown as single dots and mean. Diabetic values were defined as BGCs ≥ 300 mg/dL (gray shading) in (A). The dotted horizontal line represents the limit of quantification given by the ELISA kit (100 pg/mL) in (B). Correlation between the plasma C-peptide values and BGCs in mice treated with aCD3/vehicle (n=11) (C) and in mice treated with the aCD3/ACT-777991 combination (n=9) (D); Pearson correlation coefficient test.


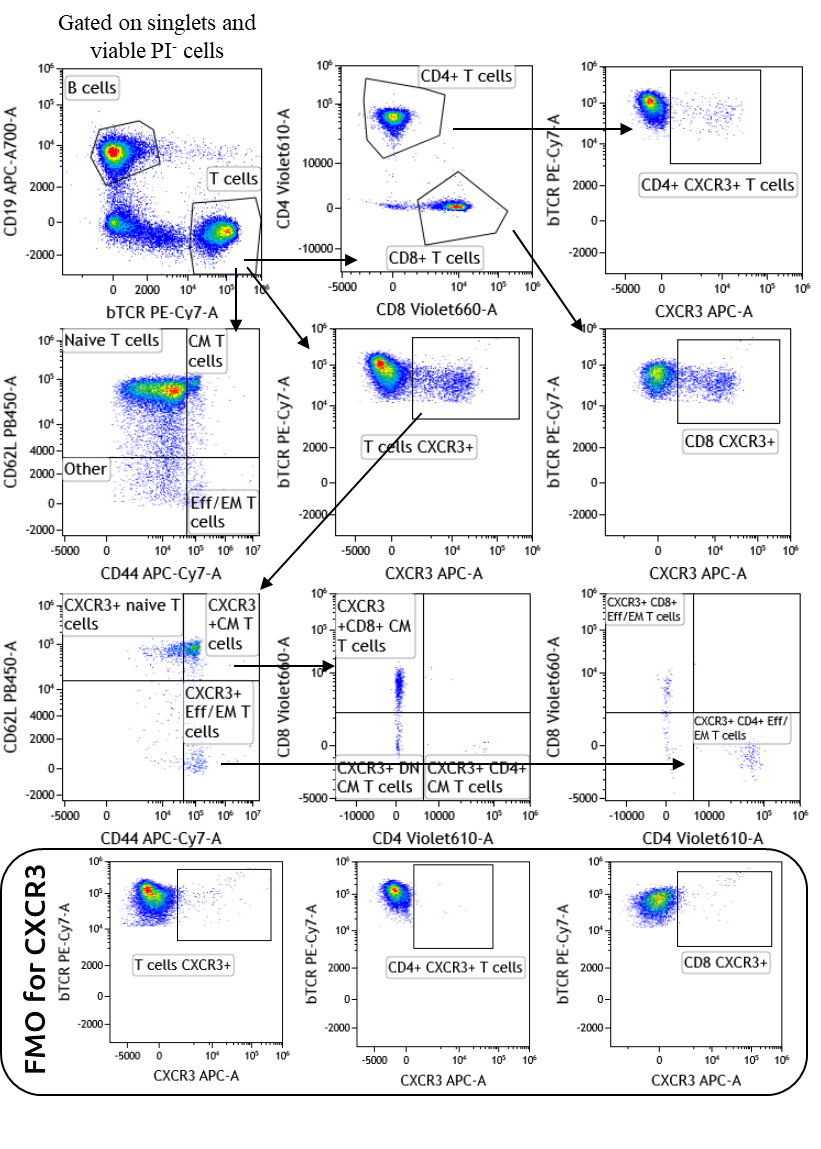


**Supplementary Figure 4**: **Gating strategy for blood immunophenotyping.** C57BL/6 mice were injected intravenously (i.v.) on Days 0, 1, and 2 with 3 µg of anti-mouse CD3ε or with isotype antibody. Blood samples were collected via tail vein puncture 24 h after the last i.v. injection and analyzed by flow cytometry. The gating strategy depicts representative plots from an isotype-treated mouse. Viable cell population was first gated based on forward scatter (FSC) versus side scatter (SSC), excluding doublets and dead cells (propidium iodide [PI]-positive cells). Among viable cells, the following cell populations were gated: B-cells (CD19^+^, **β**TCR^+^), T-cells (CD19^-^, **β**TCR^+^). Among T-cells, the following cell populations were gated: CD4^+^ T-cells (CD19^-^, **β**TCR^+^, CD4^+^), CD8^+^ T-cells (CD19^-^, **β**TCR^+^, CD8^+^), naïve T-cells (CD19^-^, **β**TCR^+^, CD62L^+^,CD44^-^), central memory (CM) T-cells (CD19^-^, **β**TCR^+^, CD62L^+^,CD44^+^), effector/effector memory (Eff/EM) T-cells (CD19^-^, **β**TCR^+^, CD62L, CD44^+^), CXCR3^+^ T-cells (CD19^-^, **β**TCR^+^, CXCR3^+^). CXCR3^+^ cells among the different T-cell subsets were defined based on the fluorescence minus one (FMO) control for CXCR3.
